# Sexual and reproductive health implementation research in humanitarian contexts: a scoping review

**DOI:** 10.1186/s12978-024-01793-2

**Published:** 2024-05-13

**Authors:** Alexandra Norton, Hannah Tappis

**Affiliations:** 1 Duke University School of Medicine, 40 Duke Medicine Circle, Durham, NC 27710 USA; 2 Johns Hopkins Bloomberg School of Public Health, 615 N. Wolfe St, Baltimore, MD 21205 USA

**Keywords:** Sexual and reproductive health, Humanitarian settings, Implementation research

## Abstract

**Background:**

Meeting the health needs of crisis-affected populations is a growing challenge, with 339 million people globally in need of humanitarian assistance in 2023. Given one in four people living in humanitarian contexts are women and girls of reproductive age, sexual and reproductive health care is considered as essential health service and minimum standard for humanitarian response. Despite growing calls for increased investment in implementation research in humanitarian settings, guidance on appropriate methods and analytical frameworks is limited.

**Methods:**

A scoping review was conducted to examine the extent to which implementation research frameworks have been used to evaluate sexual and reproductive health interventions in humanitarian settings. Peer-reviewed papers published from 2013 to 2022 were identified through relevant systematic reviews and a literature search of Pubmed, Embase, PsycInfo, CINAHL and Global Health databases. Papers that presented primary quantitative or qualitative data pertaining to a sexual and reproductive health intervention in a humanitarian setting were included.

**Results:**

Seven thousand thirty-six unique records were screened for inclusion, and 69 papers met inclusion criteria. Of these, six papers explicitly described the use of an implementation research framework, three citing use of the Consolidated Framework for Implementation Research. Three additional papers referenced other types of frameworks used in their evaluation. Factors cited across all included studies as helping the intervention in their presence or hindering in their absence were synthesized into the following Consolidated Framework for Implementation Research domains: Characteristics of Systems, Outer Setting, Inner Setting, Characteristics of Individuals, Intervention Characteristics, and Process.

**Conclusion:**

This review found a wide range of methodologies and only six of 69 studies using an implementation research framework, highlighting an opportunity for standardization to better inform the evidence for and delivery of sexual and reproductive health interventions in humanitarian settings. Increased use of implementation research frameworks such as a modified Consolidated Framework for Implementation Research could work toward both expanding the evidence base and increasing standardization.

**Plain English summary:**

Three hundred thirty-nine million people globally were in need of humanitarian assistance in 2023, and meeting the health needs of crisis-affected populations is a growing challenge. One in four people living in humanitarian contexts are women and girls of reproductive age, and provision of sexual and reproductive health care is considered to be essential within a humanitarian response. Implementation research can help to better understand how real-world contexts affect health improvement efforts. Despite growing calls for increased investment in implementation research in humanitarian settings, guidance on how best to do so is limited. This scoping review was conducted to examine the extent to which implementation research frameworks have been used to evaluate sexual and reproductive health interventions in humanitarian settings. Of 69 papers that met inclusion criteria for the review, six of them explicitly described the use of an implementation research framework. Three used the Consolidated Framework for Implementation Research, a theory-based framework that can guide implementation research. Three additional papers referenced other types of frameworks used in their evaluation. This review summarizes how factors relevant to different aspects of implementation within the included papers could have been organized using the Consolidated Framework for Implementation Research. The findings from this review highlight an opportunity for standardization to better inform the evidence for and delivery of sexual and reproductive health interventions in humanitarian settings. Increased use of implementation research frameworks such as a modified Consolidated Framework for Implementation Research could work toward both expanding the evidence base and increasing standardization.

**Supplementary Information:**

The online version contains supplementary material available at 10.1186/s12978-024-01793-2.

## Background

Over the past few decades, the field of public health implementation research (IR) has grown as a means by which the real-world conditions affecting health improvement efforts can be better understood. Peters et al. put forward the following broad definition of IR for health: “IR is the scientific inquiry into questions concerning implementation – the act of carrying an intention into effect, which in health research can be policies, programmes, or individual practices (collectively called interventions)” [1].

As IR emphasizes real-world circumstances, the context within which a health intervention is delivered is a core consideration. However, much IR implemented to date has focused on higher-resource settings, with many proposed frameworks developed with particular utility for a higher-income setting [2]. In recognition of IR’s potential to increase evidence across a range of settings, there have been numerous reviews of the use of IR in lower-resource settings as well as calls for broader use [3, 4]. There have also been more focused efforts to modify various approaches and frameworks to strengthen the relevance of IR to low- and middle-income country settings (LMICs), such as the work by Means et al. to adapt a specific IR framework for increased utility in LMICs [2].

Within LMIC settings, the centrality of context to a health intervention’s impact is of particular relevance in humanitarian settings, which present a set of distinct implementation challenges [5]. Humanitarian responses to crisis situations operate with limited resources, under potential security concerns, and often under pressure to relieve acute suffering and need [6]. Given these factors, successful implementation of a particular health intervention may require different qualities than those that optimize intervention impact under more stable circumstances [7]. Despite increasing recognition of the need for expanded evidence of health interventions in humanitarian settings, the evidence base remains limited [8]. Furthermore, despite its potential utility, there is not standardized guidance on IR in humanitarian settings, nor are there widely endorsed recommendations for the frameworks best suited to analyze implementation in these settings.

Sexual and reproductive health (SRH) is a core aspect of the health sector response in humanitarian settings [9]. Yet, progress in addressing SRH needs has lagged far behind other services because of challenges related to culture and ideology, financing constraints, lack of data and competing priorities [10]. The Minimum Initial Service Package (MISP) for SRH in Crisis Situations is the international standard for the minimum set of SRH services that should be implemented in all crisis situations [11]. However, as in other areas of health, there is need for expanded evidence for planning and implementation of SRH interventions in humanitarian settings. Recent systematic reviews of SRH in humanitarian settings have focused on the effectiveness of interventions and service delivery strategies, as well as factors affecting utilization, but have not detailed whether IR frameworks were used [12–15]. There have also been recent reviews examining IR frameworks used in various settings and research areas, but none have explicitly focused on humanitarian settings [2, 16].

Given the need for an expanded evidence base for SRH interventions in humanitarian settings and the potential for IR to be used to expand the available evidence, a scoping review was undertaken. This scoping review sought to identify IR approaches that have been used in the last ten years to evaluate SRH interventions in humanitarian settings.

This review also sought to shed light on whether there is a need for a common framework to guide research design, analysis, and reporting for SRH interventions in humanitarian settings and if so, if there are any established frameworks already in use that would be fit-for-purpose or could be tailored to meet this need.

## Methods

The Preferred Reporting Items for Systematic Reviews and Meta-Analyses (PRISMA) extension for scoping reviews was utilized to guide the elements of this review [17]. The review protocol was retrospectively registered with the Open Science Framework* (*https://osf.io/b5qtz*).*

### Search strategy

A two-fold search strategy was undertaken for this review, which covered the last 10 years (2013–2022). First, recent systematic reviews pertaining to research or evaluation of SRH interventions in humanitarian settings were identified through keyword searches on PubMed and Google Scholar. Four relevant systematic reviews were identified [12–15] Table 1.

**Table 1:** Relevant systematic reviews

Author (Year)	# of Papers Included	Timeframe	Types of Papers	Types of Studies	Other
Casey (2015) [12]	36	2004–2013	Peer-reviewed literature only	Quantitative evaluations	
Warren (2015) [15]	15	1980–2013	Peer-reviewed and grey literature	Quantitative evaluations	
Singh (2018) [14]	29	1980–2017	Peer-reviewed literature only	Quantitative evaluations	
Singh (2018) [13]	23	1980–2017	Peer-reviewed literature only	Quantitative and qualitative studies	Focus on service utilization

Second, a literature search mirroring these reviews was conducted to identify relevant papers published since the completion of searches for the most recent review (April 2017). Additional file 1 includes the search terms that were used in the literature search [see Additional file 1].

The literature search was conducted for papers published from April 2017 to December 2022 in the databases that were searched in one or more of the systematic reviews: PubMed, Embase, PsycInfo, CINAHL and Global Health. Searches were completed in January 2023 Table 2.

**Table 2:** Inclusion and exclusion criteria

	Included	Excluded
Topic^a^	Sexual and reproductive health interventions	All other health interventions
Population	Crisis-affected populations	Populations not affected by armed conflict or natural disaster
Crisis Phase	Studies conducted in the acute, chronic, and early recovery phases of humanitarian crises	Studies conducted solely during crisis preparedness or after crisis stabilization
Country^b^	Low- and middle-income countries	High income countries
Type of Data	Primary quantitative or qualitative data	Papers with only secondary data
Type of Publication	Primary quantitative and qualitative research studies published in peer-reviewed journals	Letters, editorials, commentaries, papers with no specific health intervention, papers with no primary data (including review papers), grey literature
Date of Publication	January 2013—December 2022	Before 2013
Language	English	Languages other than English

^a^SRH interventions defined as programs or initiatives addressing SRH needs and services outlined in the Inter-Agency Field Manual on Reproductive Health in Humanitarian settings, which includes interventions in the following technical areas: adolescent sexual and reproductive health, contraception, comprehensive abortion care, maternal and newborn health, gender-based violence, HIV, and sexually transmitted infections [97]

^b^World Bank 2012 classification used for distinguishing country classifications

### Screening

Two reviewers screened each identified study for alignment with inclusion criteria. Studies in the four systematic reviews identified were considered potentially eligible if published during the last 10 years. These papers then underwent full-text review to confirm satisfaction of all inclusion criteria, as inclusion criteria were similar but not fully aligned across the four reviews.

Literature search results were exported into a citation manager (Covidence), duplicates were removed, and a step-wise screening process for inclusion was applied. First, all papers underwent title and abstract screening. The remaining papers after abstract screening then underwent full-text review to confirm satisfaction of all inclusion criteria. Title and abstract screening as well as full-text review was conducted independently by both authors; disagreements after full-text review were resolved by consensus.

### Data extraction and synthesis

The following content areas were summarized in Microsoft Excel for each paper that met inclusion criteria: publication details including author, year, country, setting [rural, urban, camp, settlement], population [refugees, internally displaced persons, general crisis-affected], crisis type [armed conflict, natural disaster], crisis stage [acute, chronic], study design, research methods, SRH intervention, and intervention target population [specific beneficiaries of the intervention within the broader population]; the use of an IR framework; details regarding the IR framework, how it was used, and any rationale given for the framework used; factors cited as impacting SRH interventions, either positively or negatively; and other key findings deemed relevant to this review.

As the focus of this review was on the approach taken for SRH intervention research and evaluation, the quality of the studies themselves was not assessed.

## Results

**Fig. 1:**
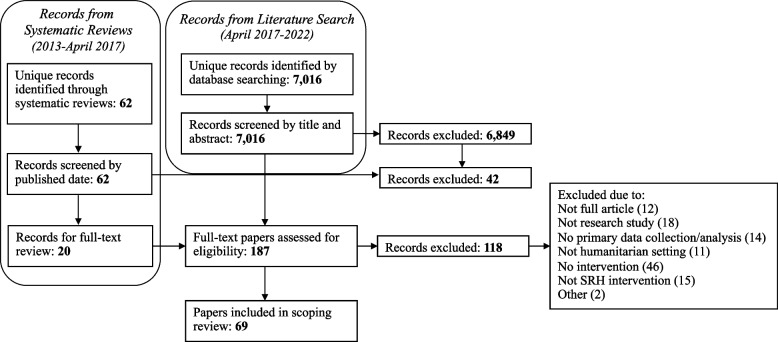
Flow chart of paper identification

Twenty papers underwent full-text review due to their inclusion in one or more of the four systematic reviews and meeting publication date inclusion criteria. The literature search identified 7,016 unique papers. After full-text screening, 69 met all inclusion criteria and were included in the review. Figure 1 illustrates the search strategy and screening process.

Papers published in each of the 10 years of the review timeframe (2013–2022) were included. 29% of the papers originated from the first five years of the time frame considered for this review, with the remaining 71% papers coming from the second half. Characteristics of included publications, including geographic location, type of humanitarian crisis, and type of SRH intervention, are presented in Table 3.

**Table 3:** Characteristics of included papers (*n* = 69)

	% of Papers	# of Papers	Papers
**Region**			
Asia and the Pacific	33%	23	Castillo et al., Corna et al., Devine et al., Draiko et al., Edmond et al. [36], Edmond et al. [37], Edmond et al. [23], Foster et al., Gibbs et al., Guan et al., Khan et al., Myers et al., Perera et al., Persson et al., Phanwichatkul et al., Santo et al., Sarker et al., Stevens et al., Tanabe et al., Thommesen et al., Tousaw et al. [51], Tousaw et al. [52], Turner et al
Latin America and the Caribbean	1%	1	Logie et al. [19]
Middle East and North Africa	10%	7	Kabakian-Khasholian et al., Lilleston et al., Morris et al., Nasir et al., Vries et al., West et al., Yankah et al
Southern and Eastern Africa	29%	20	Adam [21], Adam [26], Adam et al., Amsalu et al. [61], Amsalu et al. [65], Bakesiima et al., Casey et al., Doocy et al., Ferreyra et al., Glass et al., Greene et al., Klabbers et al., Logie et al. [70], Mugo et al., Muuo et al., O’Connell et al., O’Laughlin et al. [25], O’Laughlin et al. [20], Sami et al. [71], Sami et al. [64]
West and Central Africa	19%	13	Anibueze et al., Awasom-Fru et al., Berg et al., Bolan et al., Castle et al., Deitch et al., Gupta et al., Ho and Wheeler, Hossain et al., Hynes et al., Jarrett et al., Le Roux et al., Vaillant et al
Multiple	7%	5	James et al., Orya et al., Smith et al., Tran et al. [58], Tran et al. [74]
**Setting**			
Camp	20%	14	Adam [21], Adam [26], Adam et al., Anibueze et al., Corna et al., Greene et al., Logie et al. [70], Muuo et al., Persson et al., Sami et al. [71], Sami et al. [64], Sarker et al., Turner et al., West et al
Camp/rural	4%	3	Casey et al., Devine et al., Tran et al. [58]
Camp/rural/urban	3%	2	Nasir et al., Tran et al. [74]
Camp/urban	1%	1	Smith et al
Rural	45%	31	Bolan et al., Castle et al., Deitch et al., Draiko et al., Edmond et al. [36], Edmond et al. [37], Edmond et al. [23], Ferreyra et al., Foster et al., Guan et al., Gupta et al., Ho and Wheeler, Hossain et al., Hynes et al., Jarrett et al., Khan et al., Klabbers et al., Le Roux et al., Lilleston et al., Logie et al. [19], Myers et al., O’Connell et al., Orya et al., Perera et al., Phanwichatkul et al., Santo et al., Stevens et al., Tanabe et al., Thommesen et al., Tousaw et al. [52], Vaillant et al
Rural/urban	3%	2	Castillo et al., Morris et al
Settlement	4%	3	Bakesiima et al., O’Laughlin et al. [25], O’Laughlin et al. [20]
Urban	14%	10	Amsalu et al. [61], Amsalu et al. [65], Berg et al., Doocy et al., Gibbs et al., Glass et al., Mugo et al., Tousaw et al. [51], Vries et al., Yankah et al
Not specified	4%	3	Awasom-Fru et al., James et al., Kabakian-Khasholian et al
**Crisis Type**			
Armed Conflict	94%	65	Adam [21], Adam [26], Adam et al., Amsalu et al. [61], Amsalu et al. [65], Anibueze et al., Awasom-Fru et al., Bakesiima et al., Berg et al., Bolan et al., Casey et al., Castle et al., Corna et al., Deitch et al., Devine et al., Draiko et al., Edmond et al. [36], Edmond et al. [37], Edmond et al. [23], Ferreyra et al., Foster et al., Gibbs et al., Glass et al., Greene et al., Guan et al., Gupta et al., Ho and Wheeler, Hossain et al., Hynes et al., James et al., Jarrett et al., Kabakian-Khasholian et al., Khan et al., Klabbers et al., Le Roux et al., Lilleston et al., Logie et al. [70], Morris et al., Mugo et al., Muuo et al., Nasir et al., O’Connell et al., O’Laughlin et al. [25], O’Laughlin et al. [20], Orya et al., Perera et al., Persson et al., Phanwichatkul et al., Sami et al. [71], Sami et al. [64], Santo et al., Sarker et al., Smith et al., Stevens et al., Tanabe et al., Thommesen et al., Tousaw et al. [51], Tousaw et al. [52], Tran et al. [58], Tran et al. [74], Turner et al., Vaillant et al., Vries et al., West et al., Yankah et al
Armed Conflict/Natural Disaster	1%	1	Doocy et al
Natural disaster	4%	3	Castillo et al., Logie et al. [19], Myers et al
**SRH Technical Area**			
Abortion	9%	6	Deitch et al., Foster et al., Persson et al., Tousaw et al. [51], Tousaw et al. [52], Tran et al. [74]
Contraception	12%	8	Adam [26], Anibueze et al., Bakesiima et al., Casey et al., Castle et al., Ho and Wheeler, Morris et al., West et al
Contraception and abortion	1%	1	Tran et al. [58]
Gender-based violence	20%	14	Gibbs et al., Glass et al., Greene et al., Gupta et al., Hossain et al., James et al., Le Roux et al., Lilleston et al., Logie et al. [70], Muuo et al., Smith et al., Tanabe et al., Vaillant et al., Yankah et al
HIV	6%	4	Ferreyra et al., Klabbers et al., O’Laughlin et al. [25], O’Laughlin et al. [20]
HIV and sexually transmitted infections	1%	1	Logie et al. [19]
Maternal and newborn health	45%	31	Adam [21], Amsalu et al. [61], Amsalu et al. [65], Berg et al., Bolan et al., Castillo et al., Corna et al., Devine et al., Doocy et al., Draiko et al., Edmond et al. [36], Edmond et al. [37], Edmond et al. [23], Guan et al., Hynes et al., Jarrett et al., Kabakian-Khasholian et al., Khan et al., Mugo et al., Nasir et al., Orya et al., Perera et al., Phanwichatkul et al., Sami et al. [71], Sami et al. [64], Santo et al., Sarker et al., Stevens et al., Thommesen et al., Turner et al., Vries et al
SRH (general)	6%	4	Adam et al., Awasom-Fru et al., Myers et al., O’Connell et al

A wide range of study designs and methods were used across the papers, with both qualitative and quantitative studies well represented. Twenty-six papers were quantitative evaluations [18–43], 17 were qualitative [44–60], and 26 used mixed methods [61–86]. Within the quantitative evaluations, 15 were observational, while five were quasi-experimental, five were randomized controlled trials, and one was an economic evaluation. Study designs as classified by the authors of this review are summarized in Table 4.

**Table 4:** Study design and methods

	% of Papers	# of Papers	Papers
**Quantitative**			
Economic evaluation	1.4%	1	Devine et al
Observational (cohort)	4.3%	3	Jarrett et al., Logie et al. [19], O'Laughlin et al. [20]
Observational (cross-sectional)	5.8%	4	Adam [21], Adam et al., Edmond et al. [23], Nasir et al
Observational (pilot)	1.4%	1	O'Laughlin et al. [25]
Observational (pre-post)	8.7%	6	Adam [26], Casey et al., Corna et al., Glass et al., James et al., Le Roux et al
Observational (retrospective descriptive)	1.4%	1	Morris et al
Quasi-experimental	7.2%	5	Anibueze et al., Doocy et al., Draiko et al., Edmond et al. [36], Edmond et al. [37]
Randomized controlled trial	7.2%	5	Bakesiima et al., Greene et al., Gupta et al., Hossain et al., Vaillant et al
**Mixed Methods**			
Observational (cohort) with qualitative	1.4%	1	Muuo et al
Observational (cross-sectional) with qualitative	4.3%	3	Amsalu et al. [61], Myers et al., Santo et al
Observational (other) with qualitative	1.4%	1	Sami et al. [64]
Observational (pre-post) with qualitative	14.5%	10	Amsalu et al. [65], Berg et al., Castillo et al., Foster et al., Guan et al., Logie et al. [70], Sami et al. [71], Smith et al., Stevens et al., Tran et al. [74]
Observational (retrospective descriptive) with qualitative	10.1%	7	Castle et al., Deitch et al., Ferreyra et al., Ho and Wheeler, Klabbers et al., Turner et al., Vries et al
Pilot RCT with qualitative	2.9%	2	Bolan et al., Khan et al
Quasi-experimental with qualitative	1.4%	1	Hynes et al
RCT with qualitative	1.4%	1	Gibbs et al
**Qualitative**			
Case study	1.4%	1	Thommesen et al
In-depth interviews	14.5%	10	Awasom-Fru et al., Kabakian-Khasholian et al., Lilleston et al., Mugo et al., Persson et al., Phanwichatkul et al., Sarker et al., Tousaw et al. [51], Tousaw et al. [52], West et al
Other qualitative	8.7%	6	O’Connell et al., Orya et al., Perera et al., Tanabe et al., Tran et al. [58], Yankah et al

Six papers (9%) explicitly cited use of an IR framework. Three of these papers utilized the *Consolidated Framework for Implementation Research* (CFIR) [51, 65, 70]. The CFIR is a commonly used determinant framework that—in its originally proposed form in 2009—is comprised of five domains, each of which has constructs to further categorize factors that impact implementation. The CFIR domains were identified as core content areas influencing the effectiveness of implementation, and the constructs within each domain are intended to provide a range of options for researchers to select from to “guide diagnostic assessments of implementation context, evaluate implementation progress, and help explain findings.” [87] To allow for consistent terminology throughout this review, the original 2009 CFIR domains and constructs are used.

Guan et al. conducted a mixed methods study to assess the feasibility and effectiveness of a neonatal hepatitis B immunization program in a conflict-affected rural region of Myanmar. Guan et al. report mapping data onto the CFIR as a secondary analysis step. They describe that “CFIR was used as a comprehensive meta-theoretical framework to examine the implementation of the Hepatitis B Virus vaccination program,” and implementation themes from multiple study data sources (interviews, observations, examination of monitoring materials) were mapped onto CFIR constructs. They report their results in two phases – Pre-implementation training and community education, and Implementation – with both anchored in themes that they had mapped onto CFIR domains and constructs. All but six constructs were included in their analysis, with a majority summarized in a table and key themes explored further in the narrative text. They specify that most concerns were identified within the Outer Setting and Process domains, while elements identified within the Inner Setting domain provided strength to the intervention and helped mitigate against barriers [70].

Sarker et al. conducted a qualitative study to assess provision of maternal, newborn and child health services to Rohingya refugees residing in camps in Cox’s Bazar, Bangladesh. They cite using CFIR as a guide for thematic analysis, applying it after a process of inductive and deductive coding to index these codes into the CFIR domains. They utilized three of the five CFIR domains (Outer Setting, Inner Setting, and Process), stating that the remaining two domains (Intervention Characteristics and Characteristics of Individuals) were not relevant to their analysis. They then proposed two additional CFIR domains, Context and Security, for use in humanitarian contexts. In contrast to Guan et al., CFIR constructs are not used nor mentioned by Sarker et al., with content under each domain instead synthesized as challenges and potential solutions. Regarding the CFIR, Sarker et al. write, “The CFIR guided us for interpretative coding and creating the challenges and possible solutions into groups for further clarification of the issues related to program delivery in a humanitarian crisis setting.” [51]

Sami et al. conducted a mixed methods case study to assess the implementation of a package of neonatal interventions at health facilities within refugee and internally displaced persons camps in South Sudan. They reference use of the CFIR earlier in the study than Sarker et al., basing their guides for semi-structured focus group discussions on the CFIR framework. They similarly reference a general use of the CFIR framework as they conducted thematic analysis. Constructs are referenced once, but they do not specify whether their application of the CFIR framework included use of domains, constructs, or both. This may be in part because they then applied an additional framework, the World Health Organization (WHO) Health System Framework, to present their findings. They describe a nested approach to their use of these frameworks: “Exploring these [CFIR] constructs within the WHO Health Systems Framework can identify specific entry points to improve the implementation of newborn interventions at critical health system building blocks.” [65]

Three papers cite use of different IR frameworks. Bolan et al. utilized the Theoretical Domains Framework in their mixed methods feasibility study and pilot cluster randomized trial evaluating pilot use of the Safe Delivery App by maternal and newborn health workers providing basic emergency obstetric and newborn care in facilities in the conflict-affected Maniema province of the Democratic Republic of the Congo (DRC). They used the Theroetical Domains Framework in designing interview questions, and further used it as the coding framework for their analysis. Similar to the CFIR, the Theoretical Domains Framework is a determinant framework that consists of domains, each of which then includes constructs. Bolan et al. utilized the Theoretical Domains Framework at the construct level in interview question development and at the domain level in their analysis, mapping interview responses to eight of the 14 domains [83]. Berg et al. report using an “exploratory design guided by the principles of an evaluation framework” developed by the Medical Research Council to analyze the implementation process, mechanisms of impact, and outcomes of a three-pillar training intervention to improve maternal and neonatal healthcare in the conflict-affected South Kivu province of the DRC [67, 88]. Select components of this evaluation framework were used to guide deductive analysis of focus group discussions and in-depth interviews [67]. In their study of health workers’ knowledge and attitudes toward newborn health interventions in South Sudan, before and after training and supply provision, Sami et al. report use of the Conceptual Framework of the Role of Attitudes in Evidence-Based Practice Implementation in their analysis process. The framework was used to group codes following initial inductive coding analysis of in-depth interviews [72].

Three other papers cite use of specific frameworks in their intervention evaluation [19, 44, 76]. As a characteristic of IR is the use of an explicit framework to guide the research, the use of the frameworks in these three papers meets the intention of IR and serves the purpose that an IR framework would have in strengthening the analytical rigor. Castle et al. cite use of their program’s theory of change as a framework for a mixed methods evaluation of the provision of family planning services and more specifically uptake of long-acting reversible contraception use in the DRC. They describe use of the theory of change to “enhance effectiveness of [long-acting reversible contraception] access and uptake.” [76] Thommesen et al. cite use of the AAAQ (Availability, Accessibility, Acceptability and Quality) framework in their qualitative study assessing midwifery services provided to pregnant women in Afghanistan. This framework is focused on the “underlying elements needed for attainment of optimum standard of health care,” but the authors used it in this paper to evaluate facilitators and barriers to women accessing midwifery services [44]. Jarrett et al. cite use of the Centers for Disease Control and Prevention’s (CDC) Guidelines for Evaluating Public Health Surveillance Systems to explore the characteristics of a population mobility, mortality and birth surveillance system in South Kivu, DRC. Use of these CDC guidelines is cited as one of four study objectives, and commentary is included in the Results section pertaining to each criteria within these guidelines, although more detail regarding use of these guidelines or the authors’ experience with their use in the study is not provided [19].

Overall, 22 of the 69 papers either explicitly or implicitly identified IR as relevant to their work. Nineteen papers include a focus on feasibility (seven of which did not otherwise identify the importance of exploring questions concerning implementation), touching on a common outcome of interest in implementation research [89].

While a majority of papers did not explicitly or implicitly use an IR framework to evaluate their SRH intervention of focus, most identified factors that facilitated implementation when they were present or served as a barrier when absent. Sixty cite factors that served as facilitators and 49 cite factors that served as barriers, with just three not citing either. Fifty-nine distinct factors were identified across the papers.

Three of the six studies that explicitly used an IR framework used the CFIR, and the CFIR is the only IR framework that was used by multiple studies. As previously mentioned, Means et al. put forth an adaptation of the CFIR to increase its relevance in LMIC settings, proposing a sixth domain (Characteristics of Systems) and 11 additional constructs [2]. Using the expanded domains and constructs as proposed by Means et al., the 59 factors cited by papers in this review were thematically grouped into the six domains: Characteristics of Systems, Outer Setting, Inner Setting, Characteristics of Individuals, Intervention Characteristics, and Process. Within each domain, alignment with CFIR constructs was assessed for, and alignment was found with 29 constructs: eight of Means et al.’s 11 constructs, and 21 of the 39 standard CFIR constructs. Three factors did not align with any construct (all fitting within the Outer Setting domain), and 14 aligned with a construct label but not the associated definition. Table 5 synthesizes the mapping of factors affecting SRH intervention implementation to CFIR domains and constructs, with the construct appearing in italics if it is considered to align with that factor by label but not by definition.

**Table 5:** Factors cited as impacting SRH interventions^a^

CFIR Domain	CFIR Construct	Description of factors cited with construct	Papers including as factor that helps when present	Papers including as factor that is a barrier when absent
Characteristics of Systems	Systems architecture	Referral services and public health infrastructure	Castillo et al., Myers et al	Amsalu et al. [65], Awasom-Fru et al., Edmond et al. [23], Kabakian-Khasholian et al., Myers et al., Phanwichatkul et al., Sami et al. [64], Sarker et al
		Transportation access and infrastructure	–^b^	Castillo et al., Edmond et al. [36], Jarrett et al., Klabbers et al., Orya et al., Perera et al., Sarker et al
	Resource continuity	Disaster and disruption preparedness	Myers et al., O’Connell et al	–
		Service reliability	Edmond et al. [23]	–
		Supply chain reliability	Myers et al., O’Connell et al., Persson et al., Sarker et al	Adam [26], Devine et al., Guan et al., Sarker et al
		Sustainable source of funding	Morris et al	Tousaw et al. [51]
	Resource source	Intervention-specific funding	Guan et al	Edmond et al. [23], Sami et al. [64]
Outer Setting	Patient needs and resources	Focus and adequacy of intervention relative to community need	–	Guan et al., O’Connell et al
	Cosmopolitanism	Partnerships and coordination with NGOs and other organizations	Awasom-Fru et al., Glass et al., Guan et al., Klabbers et al., Morris et al., O’Connell et al	Castillo et al., Myers et al., Sarker et al
	*Cosmopolitanism*	Government partnerships	Castle et al., Draiko et al., Myers et al., O’Connell et al., Persson et al	Guan et al
	External policy and incentives	Governmental or other policy, or existing legal structure supportive of intervention	Persson et al	Awasom-Fru et al., Foster et al., Persson et al., Tousaw et al. [52], Yankah et al
	Community characteristics	Community and patient health literacy	–	Ho and Wheeler, Perera et al., Stevens et al
		Cultural factors affecting intervention impact	–	Gibbs et al., Klabbers et al., Muuo et al., O’Connell et al., Perera et al., Persson et al., Santo et al
		Population stability	Adam [26]	–
		Trust and acceptance of health intervention	Adam et al., Draiko et al	Adam et al., Edmond et al. [36], Edmond et al. [37], Klabbers et al., Mugo et al., Sami et al. [71], Thommesen et al., Tousaw et al. [52], West et al
	–	Security for intervention access	–	Adam [26], Gibbs et al., Jarrett et al., Muuo et al., Sami et al. [64], Tanabe et al., Thommesen et al
	–	Staff and site security	Sarker et al	Castillo et al., Edmond et al. [36], Edmond et al. [37], Gibbs et al., Klabbers et al., Mugo et al., Sami et al. [71], Sarker et al., Tanabe et al., Tran et al. [58]
	–	Other effects of conflict or crisis	–	Awasom-Fru et al
Inner Setting	Culture	Culture of provider-patient trust establishment	Adam et al., Klabbers et al., Lilleston et al., Persson et al., Phanwichatkul et al., Tanabe et al., Thommesen et al., Tousaw et al. [51], Tousaw et al. [52]	Kabakian-Khasholian et al., Phanwichatkul et al., Thommesen et al., West et al
		Confidentiality	Castle et al., Deitch et al	Kabakian-Khasholian et al., Klabbers et al., Muuo et al., Yankah et al
	Implementation climate: compatibility	Organizational support for intervention	Awasom-Fru et al., Guan et al	Awasom-Fru et al
	Implementation climate: organizational incentives and rewards	Staff retention and adequate compensation	Edmond et al. [36]	Berg et al., Bolan et al., Mugo et al., Orya et al., Sarker et al., Thommesen et al
	Implementation climate: learning climate; collective efficacy	Culture of provider and staff empowerment	Berg et al., Hynes et al., Lilleston et al., Sarker et al., Smith et al	Bolan et al
	Readiness for implementation: available resources	Adequate providers and staff to support intervention	Castle et al., Edmond et al. [36], Guan et al., Jarrett et al., Persson et al., Turner et al	Awasom-Fru et al., Edmond et al. [36], Mugo et al., Myers et al., Phanwichatkul et al., Sami et al. [71], Sami et al. [64], West et al
		Adequate space and facility infrastructure to support intervention	–	Berg et al., Bolan et al., Mugo et al., Myers et al., Persson et al., Sami et al. [71], Thommesen et al
		Availability of needed supplies	Castle et al., Hynes et al., O’Connell et al., Persson et al	Bolan et al., Devine et al., Guan et al., Mugo et al., Sami et al. [64], Thommesen et al., Tran et al. [74]
		Functional health information system	Castle et al	Amsalu et al. [65], Sami et al. [64], Sarker et al
	*Readiness for Implementation: –*	Organizational policies adapted to, conducive to or supportive of intervention	Ho and Wheeler, O’Laughlin et al. [20], Smith et al	Awasom-Fru et al., Sami et al. [71], Sami et al. [64]
	Team characteristics	Consistency in provider quality	–	Greene et al., Guan et al
		Provider sense of moral duty	Awasom-Fru et al	–
Characteristics of Individuals	Knowledge and beliefs about the intervention	Knowledge of intervention importance	Sami et al. [71]	Perera et al
	Individual stage of change	Openness of provider to intervention	Castle et al., Lilleston et al., Tanabe et al., Thommesen et al., Tran et al	Persson et al., Sami et al. [71], Sami et al. [64]
Intervention Characteristics	*Evidence strength and quality*	Community education: communication campaigns	Bolan et al., Casey et al., Ho and Wheeler, Morris et al., Muuo et al., Tousaw et al. [51]	–
		Community education: other	Bakesiima et al., Tanabe et al	Myers et al., Tousaw et al. [51]
		Mechanism for patient tracking and follow-up	Foster et al., Castle et al	Vries et al
		Provider training	Berg et al., Casey et al., Castle et al., Ho and Wheeler, Morris et al., O’Connell et al., Persson et al., Tanabe et al., Tran et al. [58], Tran et al. [74], Turner et al	Edmond et al. [37], Ho and Wheeler, Mugo et al., Sami et al. [71], Sami et al. [64], Tran et al. [74]
		Provider or staff training: indirect (train the trainer)	Amsalu et al. [65], Castillo et al	–
		Provider or staff training: refresher training	Amsalu et al. [65]	Berg et al., Guan et al
		Staff training	–	Myers et al
		Supportive supervision	Amsalu et al. [65], Casey et al., Castillo et al., Castle et al., Edmond et al. [36], Ho and Wheeler, Jarrett et al., Smith et al	Castillo et al., Tran et al. [74]
	Adaptability	Camp- or community-based, or mobile services	Adam et al., Casey et al., Ferreyra et al	–
		Home-based services	Adam [21], Adam [26], Adam et al., O’Laughlin et al. [25], Vries et al	–
		Other factor increasing accessibility	Devine et al., Klabbers et al., Logie et al. [19]	Devine et al., O’Laughlin et al. [20]
		Care provision adapted to situational needs	Berg et al., Corna et al., Doocy et al., Draiko et al., Ferreyra et al., Foster et al., Jarrett et al., Kabakian-Khasholian et al	–
		Culturally appropriate gender involvement	Hossain et al., West et al	Adam et al., Lilleston et al., Perera et al., Thommesen et al
		Intervention provider or staff from target population	Adam et al., Jarrett et al., O’Connell et al., O’Laughlin et al. [25], Orya et al., Thommesen et al	–
	Complexity	Ease of implementation	Jarrett et al	–
	*Design quality and packaging*	Appropriate intervention modality	Bolan et al., Corna et al., Ferreyra et al., James et al., Khan et al., Klabbers et al., Logie et al. [70], Morris et al., Muuo et al., Persson et al., Santo et al., Tran et al. [58], Yankah et al	Nasir et al., Santo et al
		Patient and community perception of high quality care from intervention	Castle et al., Deitch et al., Gibbs et al., Muuo et al., Perera et al., Thommesen et al., Tousaw et al. [51], Tousaw et al. [52], Turner et al	Deitch et al., Edmond et al. [36], Perera et al
		Provision of needed and appropriate supplies	Amsalu et al. [65], Berg et al., Bolan et al., O’Laughlin et al. [25], Turner et al	Amsalu et al. [65], Sami et al. [71], Tran et al. [74]
	Cost	Adequate funding of intervention	Lilleston et al	Sarker et al., Stevens et al
	Perceived scalability	Scalability	Draiko et al	–
	Perceived sustainability	Sustainability	–	Jarrett et al., Klabbers et al
Process	Planning	Community involvement in design	Draiko et al., O’Connell et al	–
		Cultural awareness during development	–	James et al
	Engaging: External change agents	Community leader engagement	Castle et al., Klabbers et al., Le Roux et al., Lilleston et al., Myers et al., Santo et al	Myers et al., O’Connell et al
	*Engaging: –*	General community engagement and mobilization	Adam et al., Draiko et al., Foster et al., Gupta et al., Ho and Wheeler, O’Connell et al., Orya et al., Tousaw et al	Edmond et al. [36], Lilleston et al., Mugo et al
	Executing	Accomplishing implementation according to plan	–	Guan et al., Jarrett et al
	Reflecting and evaluating	Incorporation of reflection	Berg et al	–

^a^construct in italics if the description is considered to align with that factor by label but not by definition

^b^ “—” denotes that no papers included that aspect of a given construct

Table 6 lists the CFIR constructs that were not found to have alignment with any factor cited by the papers in this review.

**Table 6:** CFIR Constructs without factor alignment

Domain	Construct
Characteristics of Systems	External funding agent priorities (Means et al.)
	Strategic policy alignment (Means et al.)
Outer Setting	Peer pressure
Inner Setting	Structural characteristics
	Networks and communications
	Implementation climate: tension for change
	Implementation climate: compatibility
	Implementation climate: relative priority
	Implementation climate: goals and feedback
	Readiness for implementation: leadership engagement
	Readiness for implementation: access to knowledge and information
Characteristics of Individuals	Self-efficacy
	Individual identification with organization
	Other personal attributes
Intervention Characteristics	Intervention source
	Relative advantage
	Trialability
Process	Engaging: opinion leaders
	Engaging: formally appointed internal implementation leaders
	Engaging: champions
	Decision-making (Means et al.)

## Discussion

This scoping review sought to assess how IR frameworks have been used to bolster the evidence base for SRH interventions in humanitarian settings, and it revealed that IR frameworks, or an explicit IR approach, are rarely used. All four of the systematic reviews identified with a focus on SRH in humanitarian settings articulate the need for more research examining the effectiveness of SRH interventions in humanitarian settings, with two specifically citing a need for implementation research/science [12, 13]. The distribution of papers across the timeframe included in this review does suggest that more research on SRH interventions for crisis-affected populations is taking place, as a majority of relevant papers were published in the second half of the review period. The papers included a wide range of methodologies, which reflect the differing research questions and contexts being evaluated. However, it also invites the question of whether there should be more standardization of outcomes measured or frameworks used to guide analysis and to facilitate increased comparison, synthesis and application across settings.

Three of the six papers that used an IR framework utilized the CFIR. Guan et al. used the CFIR at both a domain and construct level, Sarker et al. used the CFIR at the domain level, and Sami et al. did not specify which CFIR elements were used in informing the focus group discussion guide [51, 65, 70]. It is challenging to draw strong conclusions about the applicability of CFIR in humanitarian settings based on the minimal use of CFIR and IR frameworks within the papers reviewed, although Guan et al. provides a helpful model for how analysis can be structured around CFIR domains and constructs. It is worth considering that the minimal use of IR frameworks, and more specifically CFIR constructs, could be in part because that level of prescriptive categorization does not allow for enough fluidity in humanitarian settings. It also raises questions about the appropriate degree of standardization to pursue for research done in these settings.

The mapping of factors affecting SRH intervention implementation provides an example of how a modified CFIR framework could be used for IR in humanitarian contexts. This mapping exercise found factors that mapped to all five of the original CFIR domains as well as the sixth domain proposed by Means et al. All factors fit well within the definition for the selected domain, indicating an appropriate degree of fit between these existing domains and the factors identified as impacting SRH interventions in humanitarian settings. On a construct level, however, the findings were more variable, with one-quarter of factors not fully aligning with any construct. Furthermore, over 40% of the CFIR constructs (including the additional constructs from Means et al.) were not found to align with any factors cited by the papers in this review, also demonstrating some disconnect between the parameters posed by the CFIR constructs and the factors cited as relevant in a humanitarian context.

It is worth noting that while the CFIR as proposed in 2009 was used in this assessment, as well as in the included papers which used the CFIR, an update was published in 2022. Following a review of CFIR use since its publication, the authors provide updates to construct names and definitions to “make the framework more applicable across a range of innovations and settings.” New constructs and subconstructs were also added, for a total of 48 constructs and 19 subconstructs across the five domains [90]. A CFIR Outcomes Addendum was also published in 2022, based on recommendations for the CFIR to add outcomes and intended to be used as a complement to the CFIR determinants framework [91]. These expansions to the CFIR framework may improve applicability of the CFIR in humanitarian settings. Several constructs added to the Outer Setting domain could be of particular utility – critical incidents, local attitudes, and local conditions, each of which could help account for unique challenges faced in contexts of crisis. Sub-constructs added within the Inner Setting domain that seek to clarify structural characteristics and available resources would also be of high utility based on mapping of the factors identified in this review to the original CFIR constructs. As outcomes were not formally included in the CFIR until the 2022 addendum, a separate assessment of implementation outcomes was not undertaken in this review. However, analysis of the factors cited by papers in this review as affecting implementation was derived from the full text of the papers and thus captures content relevant to implementation determinants that is contained within the outcomes.

Given the demonstrated need for additional flexibility within an IR framework for humanitarian contexts, while not a focus of this review, it is worth considering whether a different framework could provide a better fit than the CFIR. Other frameworks have differing points of emphasis that would create different opportunities for flexibility but that do not seem to resolve the challenges experienced in applying the CFIR to a humanitarian context. As one example, the EPIS (Exploration, Preparation, Implementation, Sustainment) Framework considers the impact of inner and outer context on each of four implementation phases; while the constructs within this framework are broader than the CFIR, an emphasis on the intervention characteristics is missing, a domain where stronger alignment within the CFIR is also needed [92]. Alternatively, the PRISM (Practical, Robust Implementation and Sustainability Model) framework is a determinant and evaluation framework that adds consideration of context factors to the RE-AIM (Reach, Effectiveness, Adoption, Implementation, Maintenance) outcomes framework. It has a stronger emphasis on intervention aspects, with sub-domains to account for both organization and patient perspectives within the intervention. While PRISM does include aspects of context, external environment considerations are less robust and intentionally less comprehensive in scope, which would not provide the degree of alignment possible between the Characteristics of Systems and Outer Setting CFIR domains for the considerations unique to humanitarian environments [93].

Reflecting on their experience with the CFIR, Sarker et al. indicate that it can be a “great asset” in both evaluating current work and developing future interventions. They also encourage future research of humanitarian health interventions to utilize the CFIR [51]. The other papers that used the CFIR do not specifically reflect on their experience utilizing it, referring more generally to having felt that it was a useful tool [65, 70]. On their use of an evaluation framework, Berg et al. reflected that it lent useful structure and helped to identify aspects affecting implementation that otherwise would have gone un-noticed [67]. The remaining studies that utilized an IR framework did not specifically comment on their experience with its use [72, 83]. While a formal IR framework was not engaged by other studies, a number cite a desire for IR to contribute further detail to their findings [21, 37].

In their recommendations for strengthening the evidence base for humanitarian health interventions, Ager et al. speak to the need for “methodologic innovation” to develop methodologies with particular applicability in humanitarian settings [7]. As IR is not yet routinized for SRH interventions, this could be opportune timing for the use of a standardized IR framework to gauge its utility. Using an IR framework to assess factors influencing implementation of the MISP in initial stages of a humanitarian response, and interventions to support more comprehensive SRH service delivery in protracted crises, could lend further rigor and standardization to SRH evaluations, as well as inform strategies to improve MISP implementation over time. Based on categorizing factors identified by these papers as relevant for intervention evaluation, there does seem to be utility to a modified CFIR approach. Given the paucity of formal IR framework use within SRH literature, it would be worth conducting similar scoping exercises to assess for explicit use of IR frameworks within the evidence base for other health service delivery areas in humanitarian settings. In the interim, the recommended approach from this review for future IR on humanitarian health interventions would be a modified CFIR approach with domain-level standardization and flexibility for constructs that may standardize over time with more use. This would enable use of a common analytical framework and vocabulary at the domain level for stakeholders to describe interventions and the factors influencing the effectiveness of implementation, with constructs available to use and customize as most appropriate for specific contexts and interventions.

This review had a number of limitations. As this was a scoping review and a two-part search strategy was used, the papers summarized here may not be comprehensive of those written pertaining to SRH interventions over the past 10 years. Papers from 2013 to 2017 that would have met this scoping review’s inclusion criteria may have been omitted due to being excluded from the systematic reviews. The review was limited to papers available in English. Furthermore, this review did not assess the quality of the papers included or seek to assess the methodology used beyond examination of the use of an IR framework. It does, however, serve as a first step in assessing the extent to which calls for implementation research have been addressed, and identify entry points for strengthening the science and practice of SRH research in humanitarian settings.

With one in 23 people worldwide in need of humanitarian assistance, and financing required for response plans at an all-time high, the need for evidence to guide resource allocation and programming for SRH in humanitarian settings is as important as ever [94]. Recent research agenda setting initiatives and strategies to advance health in humanitarian settings call for increased investment in implementation research—with priorities ranging from research on effective strategies for expanding access to a full range of contraceptive options to integrating mental health and psychosocial support into SRH programming to capturing accurate and actionable data on maternal and perinatal mortality in a wide range of acute and protracted emergency contexts [95, 96]. To truly advance guidance in these areas, implementation research will need to be conducted across diverse humanitarian settings, with clear and consistent documentation of both intervention characteristics and outcomes, as well as contextual and programmatic factors affecting implementation.

## Conclusions

Implementation research has potential to increase impact of health interventions particularly in crisis-affected settings where flexibility, adaptability and context-responsive approaches are highlighted as cornerstones of effective programming. There remains significant opportunity for standardization of research in the humanitarian space, with one such opportunity occurring through increased utilization of IR frameworks such as a modified CFIR approach. Investing in more robust sexual and reproductive health research in humanitarian contexts can enrich insights available to guide programming and increase transferability of learning across settings.

### Supplementary Information

**Additional file 1**. Literature search terms: Exact search terms used in literature search, with additional detail on the methodology to determine search terms and definitions used for each component of the search

## Data Availability

The datasets analyzed during the current study are available from the corresponding author on reasonable request.

## Abbreviations

AAAQ: Availability, Accessibility, Acceptability and Quality
CDC: Centers for Disease Control and Prevention
CFIR: Consolidated Framework for Implementation Research
DRC: Democratic Republic of the Congo
EPIS: Exploration, Preparation, Implementation, Sustainment
IR: Implementation research
LMIC: Low and middle income country
MISP: Minimum Initial Service Package
PRISM: Practical, Robust Implementation and Sustainability Model
PRISMA: Preferred Reporting Items for Systematic Reviews and Meta-Analyses
RE-AIM: Reach, Effectiveness, Adoption, Implementation, Maintenance
SRH: Sexual and reproductive health
WHO: World Health Organization
