# Supplementary material for: Sexual and reproductive health implementation research in humanitarian contexts: a scoping review
Source: Reprod Health. 2024 May 13;21:64. doi: 10.1186/s12978-024-01793-2 (PMC11089709; doi:10.1186/s12978-024-01793-2)
Supplement: Supplementary file 1 — Additional file 1. Literature search terms: Exact search terms used in literature search, with additional detail on the methodology to determine search terms and definitions used for each component of the search [file 12978_2024_1793_MOESM1_ESM.docx]

**Additional file 1. Literature search terms**

**Sexual and Reproductive Health (SRH)**

“Sexual Health” OR “Reproductive Health” OR “Maternal Health” OR “Maternal Welfare” OR “Neonat*” OR “Perinatal*” OR “Pre$natal*” OR “Ante$natal*” OR “Post$natal*” OR “Post$part*” OR “Newborn” OR “Family Planning” OR “Family-planning” OR “Contracepti*” OR “Condom*” OR “Pregnan*” OR “Abort*” OR “Birth*” OR “Miscarriage*” OR “Still$b*” OR “Minimum Initial Service Package” OR “Obstetric*” OR “Gynecolog*” OR “Safe Motherhood” OR “EmO$C” OR “Safe Deliver*” OR “Sexually Transmitted Infection*” OR “Sexually Transmitted Disease*” OR “HIV” OR “Human Immunodeficiency Virus” OR “AIDS” OR “Acquired Immune Deficiency Syndrome” OR “Prevention Of Mother To Child Transmission” OR “PMTCT” OR “Vesicovaginal Fistula*” OR “Cystovaginal Fistula*” OR “Rectovaginal Fistula*” OR “Urethra Fistula*” OR “Vaginal Fistula*” OR “Urinary Tract Fistula*” OR “Adolescent Sexual Health” OR “Adolescent Reproductive Health” OR “Genital Trauma” OR “Genital Injury” OR “Vaginal Trauma” OR “Vaginal Injury” OR “Gender-Based Violence” OR “Gender Based Violence” OR “Partner Violence” OR “Family Violence” OR “Violence Against Women” OR “Domestic Violence” OR “Sexual Abuse” OR “Sex Crime” OR “Sexual Crime” OR “Sexual Violence” OR “Rape” OR “Physical Violence” OR “Partner Abuse” OR “Assault” OR “Sexual Harassment” OR “Sexual Coercion” OR “Forced Sex” OR “Sexual Exploitation” OR “Sexual Slavery”

**Humanitarian Setting**

“Disaster*” OR “Relief Work*” OR “Rescue Work*” OR “Emergenc*” OR “Mass Casualty Incident*” OR “Medical Mission*” OR “Humanitarian*” OR “Aid Work” OR “Refugee*” OR “Evacuee*” OR “Evacuated” OR “Displaced Population*” OR “Internal* Displace*” OR “Force* Displace*” OR “Forced Migrant*” OR “Forced Migration” OR “Displace* Human*” OR “Altruism” OR “War” OR “Armed Conflict*” OR “Conflict Zone*” OR “Conflict Affected*” OR “Conflict-Affected*” OR “Avalanche*” OR “Earthquake*” OR “Flood*” OR “Landslide*” OR “Tidal Wave*” OR “Tidalwave*” OR “Tsunami*” OR “Cyclonic Storm*” OR “Typhoon*” OR “Hurricane*” OR “Cyclone*” OR “Drought*” OR “Starvation*” OR “Famine*”

**Low- and Middle-Income Countries**

“Developing Countr*” OR “Asia” OR “Africa” OR “Pacific Island*” OR “Eastern Europe” OR “China” OR “Balkan Peninsula” OR “Transcaucasia” OR “Caribbean*” OR “Central America” OR “Gulf of Mexico” OR “Latin America” OR “South America” OR “Atlantic Island*” OR “Indian Ocean Island*” OR “Macau” OR “Philippines*” OR “West Indies” NOT “Japan”

**Research**

“Implementation*” OR “Random*” OR “Controlled*” OR “Cross Sectional Stud*” OR “Cross-Sectional Stud*” OR “Case-Control Stud*” OR “Case Control Stud*” OR “Cohort Stud*” OR “Pilot Stud*” OR “Control Area*” OR “Control Cohort*” OR “Control Compar*” OR “Control Condition*” OR “Control Design*” OR “Control Group*” OR “Control Intervention*” OR “Control Participant*” OR “Control Stud*” OR “Evaluation Stud*” OR “Prospective Stud*” OR “Retrospective Stud*” OR “Quasi-Experiment*” OR “Quasiexperiment*” OR “Quasirandom*” OR “Quasi Random*” OR “Quasicontrol*” OR “Quasi Control*” OR “Quasi Method*” OR “Quasi Stud*” OR “Quasi Trial*” OR “Quasi Design*” OR “Experimental Method*” OR “Experimental Stud*” OR “Experimental Trial*” OR “Experimental Design*” OR “Time Series Interrupt*” OR “Intervention*” OR “Impact*” OR “Effectiveness” OR “Efficacy” OR “Service*” OR “Outcome*” OR “Output*” OR “Treatment*” OR “Management*” OR “Program*” OR “Project*” OR “Semi-structured Interview*” OR “Semistructured Interview*” OR “Unstructured Interview*” OR “Informal Interview*” OR “In-depth Interview*” OR “Indepth Interview*” OR “Face-to-face Interview*” OR “Structured Interview*” OR “Interview Guide*” OR “Semi-structured Discussion*” OR “Semistructured Discussion*” OR “Unstructured Discussion*” OR “Informal Discussion*” OR “In-depth Discussion*” OR “Indepth Discussion*” OR “Face-to-face Discussion*” OR “Structured Discussion*” OR “Discussion Guide*” OR “Semi-structured Questionnaire*” OR “Semistructured Questionnaire*” OR “Unstructured Questionnaire*” OR “Informal Questionnaire*” OR “In-depth Questionnaire*” OR “Indepth Questionnaire*” OR “Face-to-face Questionnaire*” OR “Structured Questionnaire*” OR “Questionnaire Guide*” OR “Qualitative*” OR “Focus Group*” OR “Narration*” OR “Ethnograph*” OR “Field Work” OR “Fieldwork” OR “Key Informant*” OR “Economics” OR “Cost-Benefit Analys*” OR “Cost Control*” OR “Cost Savings” OR “Cost of Illness” OR “Cost Utilit*” OR “Cost effective*” OR “Cost-effective” OR “Cost-Utilit*”

Exact search terms were available for three of four systematic reviews, each containing the four search term categories that were relevant for this scoping review: humanitarian setting, research, low- and middle-income countries, and SRH. Search terms in the first three categories aligned across the three systematic reviews and were used for this scoping review, with the addition of “implementation” given this review’s focus on implementation research. The SRH terms from Warren et al were used for this scoping review, as this set of SRH terms was the most clearly synthesized, able to be accurately replicated, and encompassed all relevant papers identified from the other systematic reviews.

Definition used for SRH: Programs or initiatives addressing SRH needs and services outlined in the Inter-Agency Field Manual on Reproductive Health in Humanitarian settings, which includes interventions in the following technical areas: adolescent sexual and reproductive health, contraception, comprehensive abortion care, maternal and newborn health, gender-based violence, HIV, and sexually transmitted infections

Definition used for humanitarian setting (also used in two of the systematic reviews, Singh et al (2) and Warren et al): A serious disruption of the functioning of a community or a society causing widespread human, material, economic or environmental losses that exceed the ability of the affected community or society to cope using its own resources, necessitating a request to the national or international level for external assistance
